# A Plea for Gold Foil*Read before the Los Angeles County Dental Society at Los Angeles, February 21, 1922.

**Published:** 1922-08

**Authors:** G. B. Baird

**Affiliations:** Los Angeles


### A PLEA FOR GOLD FOIL*

BY G. B. BAIRD, D.D.S., LOS ANGELES

The practice of dentistry has within its bounds more complexing problems than all the other vocations put together. Every dentist with his practice is a law unto himself and meets his many problems in his own way; every practice is as varied as human nature itself, no two men see alike, no two men hear alike, consequently, no two men work or conduct their practices alike. What would be considered good service by one would be considered inferior by another, and so on.

Some operators prepare a cavity with a round bur and an excavator; many amalgam restorations look like the tooth had been hit with a mushroom bullet and the contact point had slipped beyond the gingival margin.

---

* Read before the Los Angeles County Dental Society at Los Angeles, February 21, 1922.

Few operators have a good equipment of cutting instruments, fewer still have a good stone on which to sharpen the few cutting instruments they have. We all admit that an amalgam restoration is about the simplest operation we have; and we as a profession are all willing to admit (to our patients, at least), that we are pretty good operators, almost the best in the city, and proceed to fall down at the first step—our cavity preparation. Personally, I never could prepare a Black cavity without an inverted cone bur, some chisels and angle formers; I find very little use for a round bur.

I believe that 50 per cent. of the failures in operative work is due to faulty cavity preparation, regardless of the material or method of restoration. Some of the first lessons we were taught in college was cavity preparation, let us go back now and learn our lesson well, for if we expect to give our patients good operative service, it is absolutely necessary that we start right.

It is the humble opinion of the essayist that "operative dentistry" is the foundation of the profession, and this being true, we should be very thoughtful of our operative work and study well the different principles and materials for the restoration of lost tooth structure. The ideal material naturally would be the one which will give the best service for the longest period of time and, this being true, I would like to call your attention to that material which, I think, has no equal for the restoration of lost tooth structure, namely, "gold foil." It is wonderful what can be done with this material. With a little practice, there is no limit to the skill and real art that can be developed in building gold restorations. You can almost read a man's character in his gold work, for he can express his very highest ideals.

I remember my thought as I witnessed the unveiling of the monument dedicated to Dr. G. V. Black by the National Dental Association at Chicago in 1918, and I

thought of the work that he had accomplished during his busy life and of the records that he left crowded to the last line with valuable scientific knowledge, that for years to come will benefit and bless mankind, and I thought of his life, how simple and unassuming and yet what a masterful mind his was; no explorer ever penetrated into the undiscovered country as this man did. He sailed the uncharted sea in a rudderless ship against the storms of criticisms, of envy, ignorance, malice, jealousy and hate, yet at the end of the long journey the profession on bended knee paid him homage.

When we condemn gold foil, when we discard it from our practice, we are tearing at the foundation of this monument for the real cornerstone upon which it was built was "gold foil."

I would not infer that there are not good men with good intentions who are not using gold foil, for there are, but to my mind there is no other material that is equal to gold foil. There are other materials that are handled very skillfully and that are indispensable even in the hands of the most skillful foil operators; they would not care to give them up, owing to many places and conditions where gold foil is not indicated, but I believe there are many places where foil is indicated but not used and for that reason I am here tonight to encourage a more extended use of gold foil.

I do not expect to teach you anything in the technique of handling gold foil; that comes by your own application; but to you who are interested in the study of foil I will be glad to give any information I can in conducting your study to obtain the best results.

Why should we encourage the use of gold foil? First, it is the best medium through which a student may learn to work in the mouth, he gets a training of hand and mind to work in a limited space, he learns by observation the anatomy of the oral cavity, the relation of the teeth and their function, the cleavage of the enamel and so on; then, in all fairness to ourselves and our patients, let us study this vital question. What do we seek to accomplish in a filling operation? Is it not to prevent caries from destroying the dental pulp? To permanently stop ragages of caries; to restore the lost tooth structure, and to restore the tooth to its original form, viz., normal occlusion, normal contact relation, natural contour of the tooth and a permanent restoration? With what material can we best accomplish the desired results?

### PHYSICAL PROPERTIES

The qualities most desired in a filling material for permanent operations are: indestructibility in the fluids of the mouth, adaptability to the walls of the cavity; freedom from shrinkage or expansion after having been made into a filling; resistance to attrition, and sustaining power against the force of mastication.

The qualities of minor importance are color, appearance, non-conductivity of thermal impressions and convenience of manipulation.

### GOLD

Of these qualities gold foil seems to possess those most essential in much the greatest degree; it is perfectly indestructible in the fluids of the mouth; it is perfectly adaptable to the walls of the cavity; it is free from objectionable shrinkage or expansion; its resistance to attrition is good and it sustains the force of mastication better than amalgam.

### ITS GREATEST VALUE

The greatest value of gold as a filling material lies in the fact that it may be adapted to the cavity walls with great force and is capable of immediately and permanently sustaining that force.

### ADAPTATION

Better adaptation may be obtained by gold foil than with any other material. By proper manipulation it can be built against the cavity walls with much force, gradually spreading the dentin, which is very elastic—securing a permanent gripping of the tooth to the filling, making a perfect joint that will resist the entrance of moisture or bacteria, and prevent discoloration of the tooth and afford protection to the dental pulp. It is this quality in gold with its relation to the quality of elasticity and strength of dentin or the mutual relation of these in the two substances, combined with its indestructibility that renders gold foil so pre-eminent as a filling material. No other known filling material can be so worked against the walls of a cavity as to make such use of the sustaining power of the elasticity of the dentin. I think the Great Architect of the Universe may have had this material in mind, so harmonious is the relationship of the physical qualities of these two substances in conjunction with each other.

### EXCLUSION OF FOIL

Now the question for your consideration is, are the above statements true? are they? Yes. Then why is gold foil being excluded for inferior materials and methods in the restoration of lost tooth structure? I realize there are many places and conditions under which gold foil can not be used, more places than there would have been had a good permanent gold restoration been made in the early stages of the decay.

### FOIL OPERATION

Is the cavity preparation more severe in a foil operation than for other materials? I would say, No. For amalgam you require bulk of material for strength which necessitates a deeper and wider cutting of tooth structure. For the inlay it requires more extravagant cutting of cavity walls to allow the removal of the wax model and the replacing of the cast. For the foil we can conserve tooth structure as it does not require the bulk for strength as with amalgam or the necessary wide cutting to accommodate the removal of wax, or replacement of bulk of cast gold. So we can safely say that the cavity preparation is less severe for the foil operation than either of the others. Some operators will say that "the long siege of malleting in a foil filling kills off my patients, they won't stand for it." I might say that quite an extensive filling can be malleted in 20, 30 or 40 minutes. My patients will stand for it. I will say that I have had more than one patient go to sleep during the malleting of a foil restoration; the rhythm of the mallet has a soothing effect and soon the patient is in the land of pleasant dreams. I really think that the finishing is the hardest part of a foil operation. This may be obviated to a great extent by a more careful use of the plugger in condensing and building to tooth form. After developing the technique to a certain stage of efficiency, I doubt very much if the foil operations are more severe on the patient than any other operation covering a like period of time.

A few years ago I heard this argument: In your hands you may be able to get better results with foil but not in mine. Dr. Prime answered the argument this way: "If you had used the same care in developing your technique in foil you would be able to get better results with foil, and if you want to give your patients the best service possible, which they are entitled to, you owe it to them, and to yourself especially, to study gold foil and develop your technique to the last degree of efficiency. I think the reason that a great many dentists do not consider gold foil more seriously is the impression they get in making their first filling while in college—the instructor tries to impress upon the mind of the student (and the instructor may be a poor operator himself) the great difficulty in making a gold foil restoration. This being new work for the student and naturally very difficult he immediately gets a wrong impression of gold foil that it takes years in many instances for him to overcome. This is to be regretted. He should be taught that this is a very valuable asset and that upon mastering the fundamental mechanical principles, underlying the manipulation of gold foil, he will possess an indispensable medium of accomplishment that will assure his success in the practice of his profession. I can honestly say that I prefer gold foil work to the exclusion of all other work in the category of the profession, and I will venture to say that every student of a ''gold foil club'' will tell you the identical thing.

### RESTORING TOOTH FORM

Last but not least, cohesive foil is the ideal material with which valuable tooth forms may be perfectly and beautifully reproduced. The grooves, pits, incline planes and cusps are easily reproduced; contact points restored, in all, a perfect and beautiful operation that can not possibly be attained with any other filling material. Within the scope of a 20-minute paper it is impossible to touch upon the different phases in the manipulation of goil foil, such as the different forms of gold, welding properties, lines of force, size of plugger points and its relation to the force of the blow in condensing gold, etc. There is one thing, however, that I wish to emphasize, and it's this, a hand mallet in the hand of a well-trained assistant will produce by far the best results in condensing gold, and with the least wear and tear on both patient and operator.

My purpose here is to make a plea for more extensive use of gold foil. Why are there more study clubs on foil than on any other one subject? For the reason that when a man becomes a skillful gold foil operator, he has developed a certain technique that enables him to handle other operations more skillfully.

I would be very glad to see the colleges go back to the old method of teaching gold foil and cavity preparation as they did twenty years ago, for to my mind, gold foil is the great truth in dentistry.—*The Pacific Dental Gazette.*

